# Hold it to be "safe": Anticoagulant and antiplatelet management before lumbar radiofrequency neurotomy

**DOI:** 10.1016/j.inpm.2026.100812

**Published:** 2026-08-25

**Authors:** David Hao, Jay Karri

**Affiliations:** a Department of Anesthesiology, Mass General Brigham, Harvard Medical School, Boston, MA, USA; b Harvard Medical School, Boston, MA, USA; c Departments of Orthopaedic Surgery and Anesthesiology, University of Maryland School of Medicine, Baltimore, MD, USA

## 1 "Hold it to be safe": Anticoagulant and antiplatelet management before lumbar radiofrequency neurotomy

A patient taking a direct oral anticoagulant arrives for a lumbar radiofrequency neurotomy (RFN). The medication list surfaces, and the instinct is immediate: hold the anticoagulant to be “safe”. This sentiment is difficult to argue with, because no one wishes to be unsafe. Yet, this reflex deserves scrutiny, because while it highlights concern for a periprocedural bleed, it also quietly discounts a delayed and potentially greater harm: a cardiovascular or thromboembolic event precipitated by interruption of anticoagulant and/or antiplatelet (AC/AP) therapy. In this setting, the once conventional “safe” choice rests on outdated practice patterns and may in fact prove the more harmful one.

Calling a practice “safe” shuts down disagreement, since questioning it looks cavalier. But used without nuance and specification, it conceals the most critical question: safe against *which* harms? Asked directly, the answer is that interrupting AC/AP medications raises a patient's total expected harm rather than lowering it.

## 2 Is there safety evidence supporting continuing AC/AP medications?

Periprocedural AC/AP continuation for standard interventional spine procedures offers perhaps the clearest illustration of evidence-based decision making, because both the clinical data and anatomy consistently point in the same direction. With properly performed lumbar RFN using multiplanar fluoroscopy, the cannula and electrode remain extra-spinal and do not enter the central canal or neuroforamen. As a result, any significant procedural bleeding would be expected to produce an intramuscular or subcutaneous hematoma, rather than the neuraxial hematoma that clinicians fear for its potential to compress the thecal sac and cauda equina. While this anatomical distinction is reassuring, the critical question is actually whether extraspinal probe placement can lead to clinically relevant hematoma formation that would necessitate AC/AP interruption.

This anatomical rationale is reinforced by results from multiple well-designed, prospective clinical studies. In a prospective cohort of 1936 patients across 12,723 standard spinal procedures, there were no major hemorrhagic complications in patients who continued AC/AP. Across a subset of 145 patients undergoing lumbar RFN procedures, the prevalence of hemorrhagic complications was zero, with a 95% confidence interval of 0 - 0.3 percent [1]. Notably, these patients were maintained on warfarin (32.4%), clopidogrel (38.6%), apixaban (31.7%), or rivaroxaban (4.1%). These findings extended an earlier cohort with the same result of no clinically relevant bleeding complications [2]. A systematic review applying GRADE methodology reached the congruent conclusion, albeit on low certainty evidence. Outside of those procedures requiring interlaminar epidural access, major bleeding complications from standard spinal procedures have not been reported. These available data collectively suggest an exceedingly low risk of clinically significant bleeding events with AC/AP continuation prior to lumbar RFN procedures.

What about the harm of holding these medications? Interruption of AC/AP medications for standard spinal procedures carried serious consequences of thromboembolic events, including death [3].The sharpest comparison comes from a series of more than 6000 procedures, in which ischemic complications occurred in 0.66% of cases where AC/AP agents were stopped versus 0.39% when they were continued, with no major bleeding events in either group [4]. In the larger of those cohorts, among the procedures in which AC/AP medications were stopped in accordance with guideline recommendations, nine patients suffered serious morbidities that included five non-fatal strokes, one myocardial infarction, one pulmonary embolism, and two deaths, one from a fatal stroke and one from a fatal myocardial infarction [1]. Synthesizing these and adjacent data, the International Pain and Spine Intervention Society's Patient Safety Committee places the risk of a serious cardiovascular or cerebrovascular event from ceasing warfarin for lumbar RFN at roughly 0.4 to 0.5 percent, with reported estimates spanning about 0.2 to 0.9 percent [5]. The major bleeding events of neuraxial hematomas that physicians fear did not materialize, but the subsequent strokes and infarctions that might be overlooked certainly did occur.

## 3 But don’t the guidelines recommend holding AC/AP medications?

The most common defense for holding AC/AP medications is that this practice is in accordance with guideline recommendations, and this defense deserves to be taken seriously. Two sets of consensus guidelines dominate. The multisociety guidelines led by the American Society of Regional Anesthesia (ASRA) stratify interventional procedures into low, intermediate, and high bleeding risk and repeatedly call for individualized, shared decisions [6]. The more recent interventional pain guidelines of the American Society of Interventional Pain Physicians (ASIPP), updated in 2024, do the same [7].

Both guidelines place lumbar RFN in the low-risk tier. The ASRA document moved thoracic and lumbar facet medial branch blocks and RFN, and sacroiliac joint injection, into the low-risk category in its current edition, a change prompted by the cohort data above [6]. The ASIPP guideline likewise classifies lumbar facet interventions, including medial branch blocks and RFN, together with sacroiliac joint injections and sacroiliac radiofrequency, as low risk procedures [7]. For low-risk procedures, neither guideline directs routine cessation of AC/AP agents and the interventional pain guideline states that low risk procedures may proceed at a therapeutic INR below 3.0^6^. They also explicitly permit continuation of AC/AP medications across these risk tiers [7].

The newer ASIPP guideline is blunter still about the balance of harms. Its leading recommendation states, with moderate evidence and a moderate strength of recommendation, that the thromboembolic risk of interrupting AC/AP therapy, and its associated morbidity and mortality, exceeds the risks of epidural hematoma and its sequelae, even while acknowledging that both risks are real [7]. The ASRA guideline offers the same logic that chronic pain patients may be hypercoagulable and a substantial share of acute coronary and other thromboembolic syndromes, on the order of one in ten in pooled data, follows aspirin withdrawal within the periprocedural cessation window, and that bridging in atrial fibrillation did not reduce thromboembolism and increased major bleeding in the BRIDGE trial [6,8].

The procedure-specific literature goes further than either guideline. The International Pain and Spine Intervention Society's Patient Safety Committee has issued a series of FactFinders devoted to exactly these procedures, lumbar medial branch blocks and RFN and their cervical counterparts, and each frames the reflexive hold as a myth [[3], [4], [5],^9^]. Their shared conclusion is that no clinically significant hemorrhagic complication has been reported for these procedures performed to technique, that the documented thromboembolic harm of stopping outweighs the self-limited soft-tissue bleeding risk of continuing, and that physicians should therefore consider continuation, deciding case by case.

All of these documents are candid about their limits. The ASRA panel could not grade the strength of its recommendations for want of large outcome studies, rested on limited clinical and animal data, and acknowledged that it consistently tended toward conservative recommendations. The ASIPP panel reached its statements through a modified Delphi process requiring 80 percent agreement, graded most of its evidence as low to moderate, and named the continued paucity of literature with discordant recommendations as its central limitation; its own members disagreed on how to classify transforaminal injections. None claims to define a standard of care, and all warn against mechanical application.

Put together, this means the reflexive hold is not faithful adherence to the guidelines. It is a selective reading of them, one that elevates each panel's conservative caution for genuinely high-risk procedures while passing over the procedure-specific low-risk guidance, the explicit recommendations against cessation, and the substantial treatment of the harms of discontinuation.

## 4 Blunt even when the interval is followed

Even when a clinician does follow the guideline recommended cessation intervals, the interval is a blunt instrument. Aspirin shows how often that presumption fails. In a multicenter cross-sectional study of 1111 patients scheduled for interventional pain procedures, a majority of aspirin takers, 56.4 percent, had normal platelet function on a point-of-care analyzer, while 14.2 percent of patients not taking aspirin had abnormal function [10]. For most aspirin takers, then, cessation would not change an already-normal platelet profile; it would surrender cardioprotection for no hemostatic gain, and the drug history alone proved a poor proxy for who actually had impaired platelets. This has limits. It measures platelet function in the lab, not clinical bleeding. The design is also cross-sectional and estimates of aspirin nonresponsiveness vary widely. But the direction reinforces the theme, and it adds that the cessation ritual can impose the thrombotic cost of platelet rebound while delivering no offsetting benefit.

## 5 So, why do we still stop AC/AP agents for these procedures?

A 2024 survey of interventional pain physicians found that even for low-risk procedures sizable minorities still discontinue these agents, with the P2Y12 inhibitors stopped by roughly 20-25% of respondents and the direct oral anticoagulants by more than 25%, although the survey is limited by a low response rate [11]. Why does the practice survive against the evidence and against the guidelines themselves?

Most of the reasons are structural rather than individual. The earliest guidance extended bleeding precautions developed for interlaminar and other neuraxial procedures to procedures that never enter the neuraxis, including lumbar RFN. That extension was made in good faith and ahead of direct evidence, but it set holding as the default. The reclassification of lumbar facet procedures to low risk, and the procedure-specific FactFinders that followed, are recent and have not been widely disseminated into everyday practice. The guidelines themselves sustain the habit. Several societies publish overlapping but non-identical recommendations, they diverge on specific agents such as aspirin and on how to classify procedures like transforaminal injections, and they repeatedly return the decision to individualized, case specific judgment.

At the individual level, recall biases are heavily weighed and emotionally charged. A single vivid anecdote of a patient, either treated personally or recalled from a colleague or a conference, who developed a bleeding complication after a lumbar RFN, can disproportionately shape clinical judgment. The recollection feels like strong evidence, but it is shaped by bias and is in fact among the weakest forms of evidence available. Part of the discrepancy is that the bleed is salient, immediate, and witnessed. The stroke or myocardial infarction that follows an interruption is delayed and occurs elsewhere, in an emergency department or on a neurology or neurosurgery service days later and is unlikely to be linked back to the pain physician. Because they require AC/AP medications in the first place, these patients already carry a high baseline risk for recurrent cardiovascular events, and tying any given event to a specific window remains speculative in most situations. The physician therefore accumulates bleeding anecdotes and essentially no holding-harm anecdotes, not because holding is safer, but because the feedback is biased and one-sided. Clinical experience, usually a reliable guide, miscalibrates here, which is exactly when population data should outweigh personal memory [12].

A further asymmetry is moral rather than statistical. A hemorrhage that follows an action the physician took feels like something the physician did; a thrombotic event that follows holding feels like something the disease did. The patient may be worse off in the second case, yet only the first is entered on the procedural physician's ledger.

## 6 So, what should “safety” ask?

This discussion is not an argument against clinical caution. Rather, we urge clinicians to reevaluate what deems a practice “safe”. Safe against which harm - the proximate and attributable one, or the larger delayed one? Across what horizon: the procedure suite, or the weeks afterward? And borne by whom: the physician's record, or the patient? The obligation is not simply to avoid the most immediate procedural risks, but also to minimize the patient's cumulative total harm.

There are real boundaries to acknowledge. The low-risk argument is specific to low-risk procedures. Both guidelines reserve a high-risk tier for interlaminar epidurals, spinal cord stimulator and intrathecal pump implantation, vertebral augmentation, and similar work, where the bleeding risk is real and interruption is appropriate; the systematic review likewise treated interlaminar access as the exception to its otherwise reassuring findings [3,5,6].

Cervical facet procedures are categorized one risk tier higher than their lumbar counterparts, labeled intermediate-rather than low-risk, given the proximity of several critical arteries to the target articular pillars. Therefore, considerations applied to lumbar RFN should not be extended to the cervical spine without individualized caution, even though the similar safety analyses report no clinically significant hemorrhagic complication with either anatomic location [4]. Because these procedures are elective, declining or deferring the intervention also remains a legitimate option when the balance is genuinely uncertain, as the safety committees themselves note [[3], [4], [5],^9^].

The argument is not that caution is wrong. Rather, caution misapplied to low-risk situations, and justified by an unexamined perception of “safety”, produces the very harm it seeks to prevent.

We should hold ourselves to a simple discipline. Decide by expected net harm across the full time horizon. And discount personal anecdotes in proportion to how one-sided the feedback that produced it. The next time the instinct is to hold an anticoagulant to be “safe”, the better question is safe against what.

## Attestation

The authors used Claude (Anthropic) for copyediting, phrasing suggestions, and cross-checking references against source records. The authors reviewed and verified all output and take full responsibility for the accuracy and integrity of the manuscript.

## Declaration of competing interests

We have no conflicts of interest to report.
